# Different expression of β subunits of the KCa1.1 channel by invasive and non-invasive human fibroblast-like synoviocytes

**DOI:** 10.1186/s13075-016-1003-4

**Published:** 2016-05-10

**Authors:** Zoltán Pethő, Mark R. Tanner, Rajeev B. Tajhya, Redwan Huq, Teresina Laragione, Gyorgy Panyi, Pércio S. Gulko, Christine Beeton

**Affiliations:** Department of Molecular Physiology and Biophysics, Mail Stop BCM335, Room S409A, Baylor College of Medicine, Houston, TX 77030 USA; Department of Biophysics and Cell Biology, Faculty of Medicine, University of Debrecen, Debrecen, 4032 Hungary; Interdepartmental Graduate Program in Translational Biology and Molecular Medicine, Baylor College of Medicine, Houston, TX 77030 USA; Graduate Program in Molecular Physiology and Biophysics, Baylor College of Medicine, Houston, TX 77030 USA; Division of Rheumatology, Icahn School of Medicine at Mount Sinai, New York, NY 10029 USA; Biology of Inflammation Center, Baylor College of Medicine, Houston, TX 77030 USA; Center for Drug Discovery, Baylor College of Medicine, Houston, TX 77030 USA

**Keywords:** Arthritis, Autoimmune disease, Cell migration, Electrophysiology, Patch clamp, Potassium channel, Regulatory subunit, Synovial fibroblast

## Abstract

**Background:**

Fibroblast-like synoviocytes (FLS) in rheumatoid arthritis (RA-FLS) contribute to joint inflammation and damage characteristic of the disease. RA-FLS express KCa1.1 (BK, Slo1, MaxiK, *KCNMA1*) as their major plasma membrane potassium channel. Blocking KCa1.1 reduces the invasive phenotype of RA-FLS and attenuates disease severity in animal models of RA. This channel has therefore emerged as a promising therapeutic target in RA. However, the pore-forming α subunit of KCa1.1 is widely distributed in the body, and blocking it induces severe side effects, thus limiting its value as a therapeutic target. On the other hand, KCa1.1 channels can also contain different accessory subunits with restricted tissue distribution that regulate channel kinetics and pharmacology. Identification of the regulatory subunits of KCa1.1 expressed by RA-FLS may therefore provide the opportunity for generating a selective target for RA treatment.

**Methods:**

Highly invasive RA-FLS were isolated from patients with RA, and FLS from patients with osteoarthritis (OA) were used as minimally invasive controls. The β subunit expression by FLS was assessed by quantitative reverse transcription polymerase chain reactions, Western blotting, and patch-clamp electrophysiology combined with pharmacological agents. FLS were sorted by flow cytometry on the basis of their CD44 expression level for comparison of their invasiveness and with their expression of KCa1.1 α and β subunits. β1 and β3 subunit expression was reduced with small interfering RNA (siRNA) to assess their specific role in KCa1.1α expression and function and in FLS invasiveness.

**Results:**

We identified functional β1 and β3b regulatory subunits in RA-FLS. KCa1.1 β3b subunits were expressed by 70 % of the cells and were associated with highly invasive CD44^high^ RA-FLS, whereas minimally invasive CD44^low^ RA-FLS and OA-FLS expressed either β1 subunit. Furthermore, we found that silencing the β3 but not the β1 subunit with siRNA reduced KCa1.1 channel density at the plasma membrane of RA-FLS and inhibited RA-FLS invasiveness.

**Conclusions:**

Our findings suggest the KCa1.1 channel composed of α and β3b subunits as an attractive target for the therapy of RA.

## Background

Rheumatoid arthritis (RA) is a chronic and systemic autoimmune disease that predominantly involves diarthrodial joints but can also target vital internal organs, including the heart and lungs [1–3]. In the last decades, management of RA has significantly improved with the development of new treatments; however, disease remission is rarely achieved, and most patients attain only mild to modest improvement [4]. Furthermore, current therapies significantly impair immune responses, rendering patients more susceptible to infections and cancer [5]. Therefore, novel therapeutic options that lead to pronounced improvement or remission without inducing immunosuppression are needed.

Fibroblast-like synoviocytes (FLS) in RA (RA-FLS) have been implicated in disease pathogenesis, as they exhibit a transformed, “tumor-like” phenotype with increased invasiveness and production of proteases and of various proinflammatory and proangiogenic factors [6–8]. Importantly, the ex vivo invasiveness of FLS correlates with radiographic and histologic damage and therefore with disease severity [9, 10].

Highly invasive RA-FLS and FLS from arthritic rats express the functional calcium-activated potassium channel KCa1.1 (also known as BK, Slo1, MaxiK, or *KCNMA1*) as the major K^+^ channel at their plasma membrane [11, 12]. Blocking the function of KCa1.1 pore-forming α subunits in these FLS with paxilline inhibits their invasiveness and stops disease progression in animal models of RA [11, 12]. However, paxilline is a lipophilic small molecule that blocks all KCa1.1 channels found in major organs, regardless of channel subunit composition [13–15], and can cross cell membranes as well as the blood-brain barrier. It therefore induces severe side effects, such as tremors, incontinence, and hypertension [16–18]. Despite the drawbacks of the unspecific block of KCa1.1 by paxilline, the channel remains an attractive target for therapy because the regulatory subunits of KCa1.1 have restricted tissue distribution and affect channel pharmacology [19, 20]. Four β subunits with distinct amino acid sequences have been described: β1 (KCNMB1, UniProt identifier Q16558), found in smooth muscles; β2 (KCNMB2, UniProt identifier Q9Y691), prevalent in the adrenal gland and brain; β3 (KCNMB3, UniProt identifier Q9NPA1), expressed mainly in the testis; and β4 (KCNMB4, UniProt identifier Q86W47), mostly expressed in the central nervous system [20–26]. The presence of β subunits affects the kinetics of the channel as well as the selectivity and affinity of various KCa1.1 channel modulators. Steroid compounds, such as lithocholic acid (LCA), selectively enhance KCa1.1 currents only if the channel contains the β1 subunit [27, 28]. In contrast, arachidonic acid (AA) amplifies currents in the presence of the β2 or β3 subunit but not the β1 or β4 subunit [29], whereas the scorpion toxins iberiotoxin and charybdotoxin (ChTX) fail to inhibit KCa1.1 in the presence of the β4 subunit [21]. In addition, the β1, β2, and β4 subunits modulate membrane translocation of the KCa1.1-conducting α subunit [23, 24, 30]. To date, however, no KCa1.1 β subunit has been described in RA-FLS. Our aims in this study were to determine whether RA-FLS express any accessory β subunits and whether expression of these subunits varies with the invasive potential of these cells.

## Methods

### Ethical considerations

FLS were collected and banked for research in a deidentified manner by the Feinstein Institute for Medical Research Tissue Donation Program (Manhasset, NY, USA) under the approval of the institutional review board (IRB) of the Feinstein Institute for Medical Research. All patients provided written consent to have their tissues, RNA, DNA, and cells studied. The consent forms are held by the Feinstein Institute Tissue Donation Program; the authors of this study had access only to cells and deidentified data. The IRB at Baylor College of Medicine has established that the work conducted there did not constitute human research, since the samples were already banked for research and were deidentified, and thus could not be traced back to their donors.

Rat testes were obtained from Prof. Robert M. Bryan’s laboratory (Department of Anesthesiology, Baylor College of Medicine, Houston, TX, USA). Rats were killed following American Veterinary Medical Association guidelines under an institutional animal care and use committee-approved protocol at Baylor College of Medicine. Discarded testes from healthy animals were obtained as control samples for our studies.

### Cells

FLS from 14 patients with RA (age 58 ± 10 years) and 4 patients with osteoarthritis (OA) (age 57 ± 5 years), defined according to the criteria of the American College of Rheumatology [31], were purchased from Asterand Bioscience (Detroit, MI, USA) or collected as described elsewhere [32, 33] (Table 1). Cells were used between passages 4 and 11 for all experiments. They were cultured in DMEM (Life Technologies, Grand Island, NY, USA) supplemented with 10 IU/ml penicillin, 0.1 g/ml streptomycin, 1 mM sodium pyruvate, 2 mg/ml l-glutamine, and 10 % FBS.

**Table 1:** Characteristics of the subjects who donated fibroblast-like synoviocytes for this study

Donor	Diagnosis	Sex	Ethnicity	Disease duration (years)	RF	Medications	Origin of cells	Figures showing which cells were used^a^
RA-1	RA	Female	White	3	+	NSAID	Asterand Bioscience	1a, b; 2a, b; 3a, b, d
RA-2	RA	Male	Hispanic	<1	+	Prednisone, DMARD	Asterand Bioscience	2a, b; 5a, b, c; 6b
RA-3	RA	Female	White	<1	+	NSAID	Asterand Bioscience	2a, b; 3a, b, d
RA-4	RA	Female	White	12	+	NSAID, prednisone	Asterand Bioscience	1a, b; 2a, b; 4c
RA-5	RA	Female	Hispanic	2	+	DMARD, NSAID	FITDP	1a, b; 4a, b, e, f, g; 5a, b, c
RA-6	RA	Female	White	21	+	Prednisone, DMARD	FITDP	3a, b, c, d
RA-7	RA	Female	White	30	+	Prednisone, DMARD	FITDP	2a, b; 3a, b, c, d; 4c, d, e, f
RA-8	RA	Female	White	20	+	Etanercept, prednisone	FITDP	1a, b; 2a, b
RA-9	RA	Female	White	21	+	Leflunomide, etanercept, prednisone	FITDP	1a, b; 4a, b; 5a, b, c; 6a, b
RA-10	RA	Male	White	15	+	NSAID, prednisone, adalimumab	FITDP	2a, b; 3c; 4c, d, e, f, g
RA-11	RA	Female	African American	11	+	Hydroxychloroquine, prednisone	FITDP	2a, b; 3c; 4c, d, e, f, g
RA-12	RA	Male	White	>10	+	Etanercept	FITDP	1a, b; 3c; 4a, b
RA-13	RA	Female	Hispanic	>10	+	Methotrexate, prednisone	FITDP	3a, b, c, d; 4c, d, e, f; 6a, b
RA-14	RA	Female	White	3	+	Prednisone, methotrexate	FITDP	5a, b, c; 6a, b
OA-1	OA	Female	White	12	−	None	Asterand Bioscience	4c, d, e, f
OA-2	OA	Male	White	16	−	None	Asterand Bioscience	4c, d, e, f
OA-3	OA	Male	White	25	N/A	None	FITDP	4c, d, e, f
OA-4	OA	Male	White	78	N/A	None	FITDP	4c, d, e, f

*DMARD* disease-modifying antirheumatic drug, *FITDP* Feinstein Institute Tissue Donation Program, *N/A* not available, *NSAID* non-steroidal anti-inflammatory drug, *OA* osteoarthritis, *RA* rheumatoid arthritis, *RF* rheumatoid factor

Patients with RA were 58 ± 10 years old, and patients with OA were 57 ± 5 years old

^a^Cross-references to figures in this article displaying which cells were used

### Quantitative reverse transcription-polymerase chain reactions

Total RNA, isolated from cell pellets using TRIzol reagent (Life Technologies), was reverse-transcribed with SuperScript III reverse transcriptase and random hexamer primers (Life Technologies) according to the manufacturer’s instructions. The resulting complementary DNA (cDNA) was used as a template for quantitative polymerase chain reaction (qPCR) primers (Table 2), which were designed from the National Institutes of Health qPrimerDepot (http://primerdepot.nci.nih.gov/) or designed manually and tested by PrimerBlast (http://www.ncbi.nlm.nih.gov/tools/primer-blast/). Amplicon sizes were between 70 and 250 bp. qPCRs were conducted in final volumes of 10 μl diluted cDNA (1:10) containing 4 μl of oligo forward and reverse primers (2.5 μM each) and 6 μl of iTAQ SYBR Green Supermix (Bio-Rad Laboratories, Hercules, CA, USA). Reactions were run in a ViiA™ 7 Real-Time PCR System (Life Technologies). The cycling condition were 20 seconds at 95 °C, 40 cycles at 95 °C for 1 second and 60 °C for 20 seconds, 95 °C for 15 seconds, 60 °C for 1 minute, and a gradient from 60 °C to 95 °C for 15 minutes. The results were analyzed using ViiA™ 7 software.

**Table 2:** Primers used for quantitative polymerase chain reactions

Subunit	Accession number	Sense (forward) 5′-3′	Antisense (reverse) 3′-5′	Base pair
α	NM_002247	GCTCAAGTACCTGTGGACCG	CTGGTTTGAGAGTGCCATCC	104
β1	NM_004137	CTGTACCACACGGAGGACAC	GCTCTGACCTTCTCCACGTC	107
β2a	NM_181361	ATTAAGCGTGGCTTTTGAGG	GTTGGTCCAGGGTCTCCTTT	98
β2b	NM_005832	GAGAAAGAGCAACAAAGCGG	TTAGCAAATCCCAGACATTGC	107
β3a	NM_171828	AAATCACACTTCAGGGCAGC	GCACATCTAGTGGGTCTCCA	105
β3b	NM_171829	TCTGAGTGTGAGGGGCTCTT	GCACATCTAGTGGGTCTCCA	105
β3c	NM_171830	CCATGATGGGCTTCTCAGTC	GCAGTGCAGGTCGATTCTTC	100
β3d	NM_014407	AGGGACGTGCAATATCCCTG	GAAAGGCTGTCCTGTGTCTCGT	351
β3e	NM_001163677	ACCCGTGTCTTCAGGTGTTT	TCTGTAACATCACGCTTGGGA	107
β4	NM_014505	CTGAGTCCAACTCTAGGGCG	GATTTTCTCTCTTACAGGGAGGG	96
GAPDH	NM_002046	AAGGTGAAGGTCGGAGTCAA	AATGAAGGGGTCATTGATGG	108

*GAPDH* glyceraldehyde 3-phosphate dehydrogenase

### Western blotting

RA-FLS and a rat testis were lysed in radioimmunoprecipitation assay buffer (Sigma-Aldrich, St. Louis, MO, USA) containing 1 % protease inhibitors. Protein levels were measured using the Bradford assay. Equal amounts of protein (20 μg) were loaded and separated by SDS-PAGE (Life Technologies), then transferred onto nitrocellulose membranes (Bio-Rad Laboratories) according to the manufacturer’s instructions. Blots were incubated in overnight blocking solution consisting of 4 % Blotto nonfat milk (Santa Cruz Biotechnology, Santa Cruz, CA, USA). Blots were probed using antibodies specific for the human KCa1.1 channel α and β subunits (Table 3). Each primary antibody was diluted 1:500 in blocking solution. As loading controls, we used antiactin antibodies. Overnight incubation and washing were followed by a 2-h probing with IR-680- or IR-800-labeled secondary antibodies (Table 3) for 1 h at room temperature. The membranes were washed three times for 15 minutes with PBS + 0.1 % Tween 20. Visualization was performed with a LI-COR Odyssey Scanner (LI-COR Biosciences, Lincoln, NE, USA), and data were analyzed with ImageJ software (National Institutes of Health, Bethesda, MD, USA).

**Table 3:** Characteristics of the antibodies used for this study

Target	Host	Vendor (catalog number; manufacturer location)	Conjugation	Clone	Use
Primary antibodies					
Actin	Rabbit	Sigma-Aldrich (A2066; St. Louis, MO, USA)	–	–	WB
Actin	Mouse	Sigma-Aldrich (A3853)	–	AC-40	WB
Cadherin-11	Mouse	Thermo Fisher Scientific (MA1-06306; Rockford, IL, USA)	–	16A	FC
CD44	Mouse	Abcam (ab187571; Cambridge, MA, USA)	Alexa Fluor 488	MEM85	FC, EP
KCa1.1α	Mouse	Antibodies, Inc. (NeuroMab, 75-022; UC Davis/NIH NeuroMab Facility, Davis, CA, USA)	–	L6/60	WB
KCa1.1α	Rabbit	EMD Millipore (AB5228; Billerica, MA, USA)	–	–	FC
KCa1.1β1	Rabbit	Novus Biologicals (NBP1-33484; Littleton, CO, USA)	–	–	WB
KCa1.1β2	Mouse	Antibodies, Inc. (NeuroMab, 75-087; UC Davis/NIH NeuroMab Facility)	–	N53/32	WB
KCa1.1 pan-β3	Rabbit	Abcam (ab137041)	–	EPR9543(B)	WB
KCa1.1 β3a, βc, βd, βe	Mouse	Rockland Immunochemicals (200-301-E96; Limerick, PA, USA)	–	S40B-18	WB
KCa1.1β4	Mouse	Antibodies, Inc. (NeuroMab, 75-086; UC Davis/NIH NeuroMab Facility)	–	L18A/3	WB
MMP-2	Mouse	BioLegend (634802; San Diego, CA, USA)	–	F14P4D3	FC
Podoplanin	Rat	BioLegend (337003)	Phycoerythrin	NC-08	FC
Secondary antibodies					
Mouse IgG	Goat	LI-COR Biosciences (926-32210; Lincoln, NE, USA)	Infrared-800	–	WB
Mouse IgG1	Goat	Thermo Fisher Scientific (A-21127)	Alexa Fluor 555	–	FC
Rabbit IgG	Donkey	LI-COR Biosciences (926-68023)	Infrared-680	–	WB
Rabbit IgG	Goat	Abcam (ab150079)	Alexa Fluor 647	–	FC

*EP* electrophysiology, *FC* flow cytometry, *IgG* immunoglobulin G, *KCa* calcium-activated potassium channel, *MMP* matrix metalloproteinase, *WB* Western blot analysis

### Electrophysiology

Cells were plated on glass coverslips and allowed to adhere. When indicated, cells were incubated for 15 minutes at room temperature with fluorophore-conjugated anti-CD44 antibodies (Table 3) and washed, and CD44^high^ and CD44^low^ cells were immediately assessed for K^+^ currents [34]. Total K^+^ currents were recorded using the patch-clamp technique in the whole-cell configuration as described previously [11, 12, 35]. The internal solution contained 10 mM ethylene glycol tetraacetic acid, 5 mM HEPES, and 5 μM free Ca^2+^, calculated using Maxchelator (http://maxchelator.stanford.edu/CaEGTA-TS.htm). The external solution contained 160 mM NaCl, 4.5 mM KCl, 2 mM CaCl_2_, 1 mM MgCl_2_, and 10 mM HEPES, pH 7.4. Experiments were performed at room temperature (20–22 °C). FLS cell capacitances ranged from 9 to 17 pF. Results were analyzed using IGOR Pro software (WaveMetrics, Lake Oswego, OR, USA).

### Ion channel modulators

LCA enhances KCa1.1 currents only when the channel contains the β1 subunit [28, 36, 37]. A stock of LCA (Sigma-Aldrich) was prepared in 1:10 solution of dimethyl sulfoxide (DMSO) and 70 % ethanol. AA enhances KCa1.1 currents only when the channel contains β2 or β3 subunits [29, 36]. A stock of AA (Sigma-Aldrich) was prepared in DMSO. Paxilline blocks KCa1.1 channels, regardless of β subunit expression [11, 14, 38]. A stock of paxilline (Fermentek, Jerusalem, Israel) was prepared in DMSO. ChTX blocks all KCa1.1 channels, unless they contain the β4 subunit [21, 36, 39]. A stock of ChTX (Peptides International, Louisville, KY, USA) was prepared in P6N buffer (10 mM NaHPO_4_, 0.8 % NaCl, 0.05 % Tween 20, pH 6.0) [40–42]. The stock solutions were further diluted with electrophysiology bath solution immediately before use so that final DMSO concentrations did not exceed 0.05 %.

### Flow cytometry

Detection of the α subunit of KCa1.1 and of CD44, podoplanin, cadherin 11, and intracellular matrix metalloproteinase (MMP)-2 was performed as previously described [12, 41, 43] using antibodies listed in Table 3. Cells were treated with brefeldin A (eBioscience, San Diego, CA, USA) for 6 h before detection of intracellular MMP-2. They were fixed and permeabilized with 0.5 % saponin for detection of intracellular epitopes. Data were acquired on a BD FACSCanto II flow cytometer (BD Biosciences, San Jose, CA, USA) using BD FACSDiva software and analyzed using FlowJo software (Treestar, Ashland, OR, USA). Alternatively, live CD44^high^ and CD44^low^ cells were sorted under sterile conditions using a FACSAria II flow cytometer (BD Biosciences) and immediately used for invasion assays.

### Small interfering RNA transfection

RA-FLS were kept in serum-free medium for 24 h before small interfering RNA (siRNA) treatment with DharmaFECT siRNA transfection reagent and siRNA against GAPDH, KCNMB1, or KCNMB3 (GE Dharmacon, Lafayette, CO) as described previously [12]. Cells were used 40–80 h later.

### Invasion assays

The ex vivo invasiveness of FLS was assayed in a transwell system using collagen-rich, Matrigel-coated inserts (BD Biosciences) as described elsewhere [11, 12, 44].

### Statistical analysis

We performed nonparametric one-way analysis of variance (Kruskal-Wallis test) to calculate the statistical significance of our results, followed by Dunn’s post hoc test (Prism software; GraphPad Software, La Jolla, CA, USA). *p* values less than 0.05 were considered significant.

## Results

### RA-FLS express multiple β subunits at the messenger RNA and protein levels

**Fig. 1:**
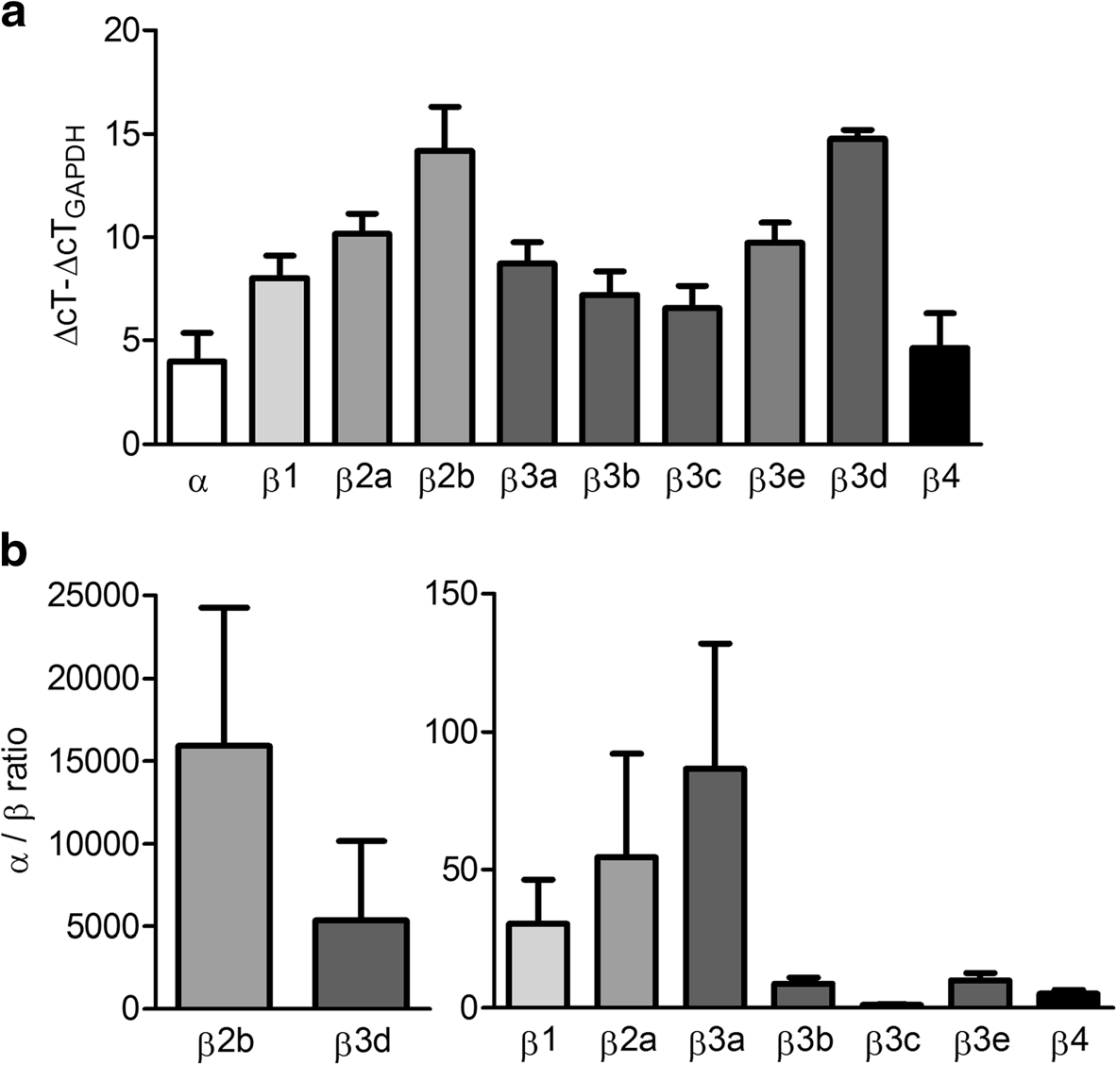
Fibroblast-like synoviocytes from patients with rheumatoid arthritis express messenger RNA of multiple KCa1.1 β subunits. **a** expression fold measurements were conducted by quantitative reverse transcription polymerase chain reaction, compared with GAPDH expression (*n* = 6 donors with 3 independent repeats). Each bar shows expression of a different KCa1.1 subunit, the letters *a*–-*e* represent different transcript variants. **b** α/β ratio showing the amount of KCa1.1 α subunits expressed for each single β subunit. Note the different scales of the *y*-axis. *ΔcT* comparative cycle threshold, *GAPDH* glyceraldehyde 3-phosphate dehydrogenase

**Fig. 2:**
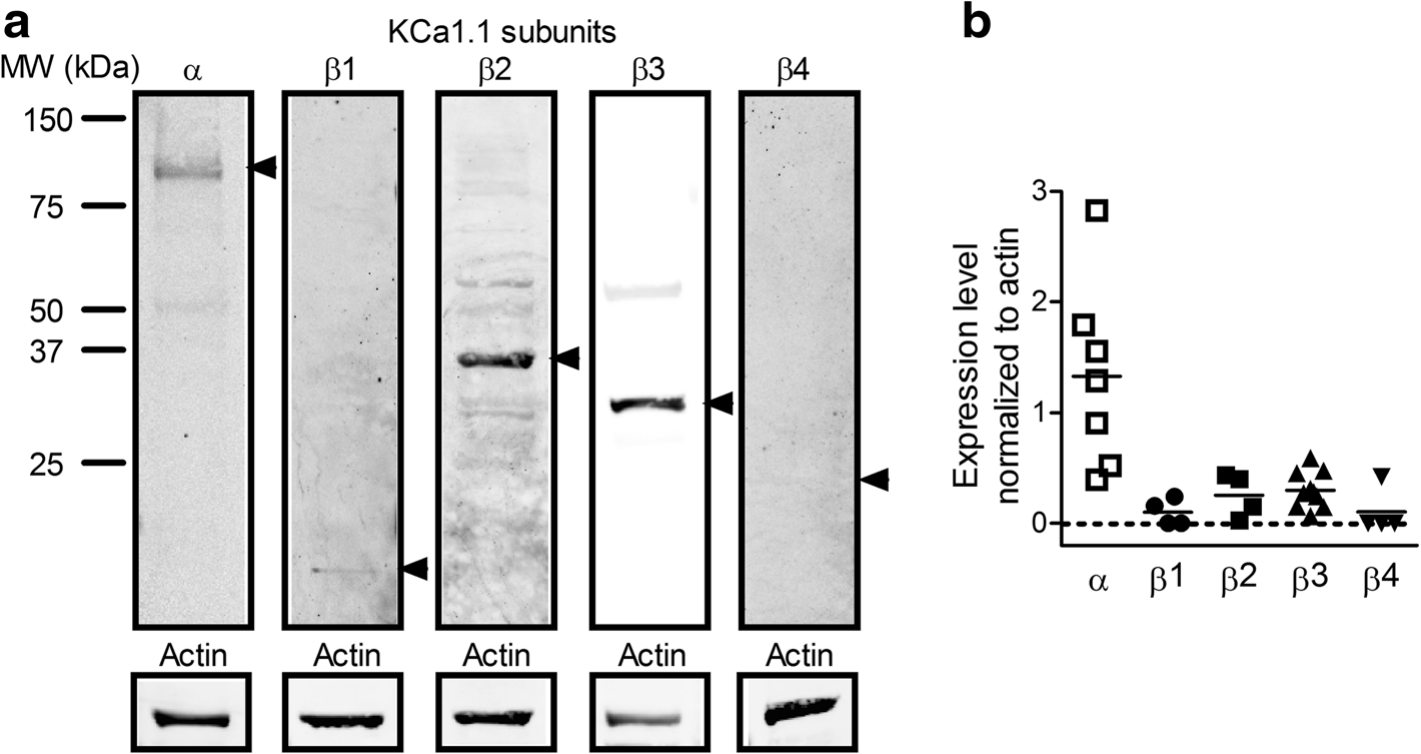
Fibroblast-like synoviocytes from patients with rheumatoid arthritis (RA-FLS) express proteins of multiple calcium-activated potassium channel KCa1.1 β subunits. **a** Representative Western blot from a gel loaded with proteins from one RA-FLS donor with different lanes probed with antibodies against different subunits of KCa1.1 (*top*) and actin (*bottom*). **b** Intensity of KCa1.1 α and β subunit protein bands normalized to actin expression levels in RA-FLS. Each symbol on the scatterplot represents results from a different donor. The *horizontal bar* represents the mean for each subunit

Analysis of messenger RNA (mRNA) levels by quantitative reverse transcription-PCR (qRT-PCR) in RA-FLS showed expression of most KCa1.1 β subunits (Fig. 1a), although the β2b and β3d subunits were barely detectable. When compared with the expression levels of the KCa1.1 pore-forming α subunit, the highest relative mRNA expression levels were found for three splice variants of the β3 subunit (β3b, β3c, and β3e) and for β4 subunits (Fig. 1b). Since mRNAs are not always translated into proteins, we used Western blotting to determine protein levels of the different β subunits. Analysis of the total cellular protein content showed variable amounts of all β subunits of KCa1.1 in different RA-FLS donors compared with the loading control actin. Whereas expression of β1, β2, and β4 was only detectable in the cell lysates from some donors, β3 subunits were consistently identified in all RA-FLS donors but one (Fig. 2).

### RA-FLS express either β1 or β3b subunits at their plasma membrane

**Fig. 3:**
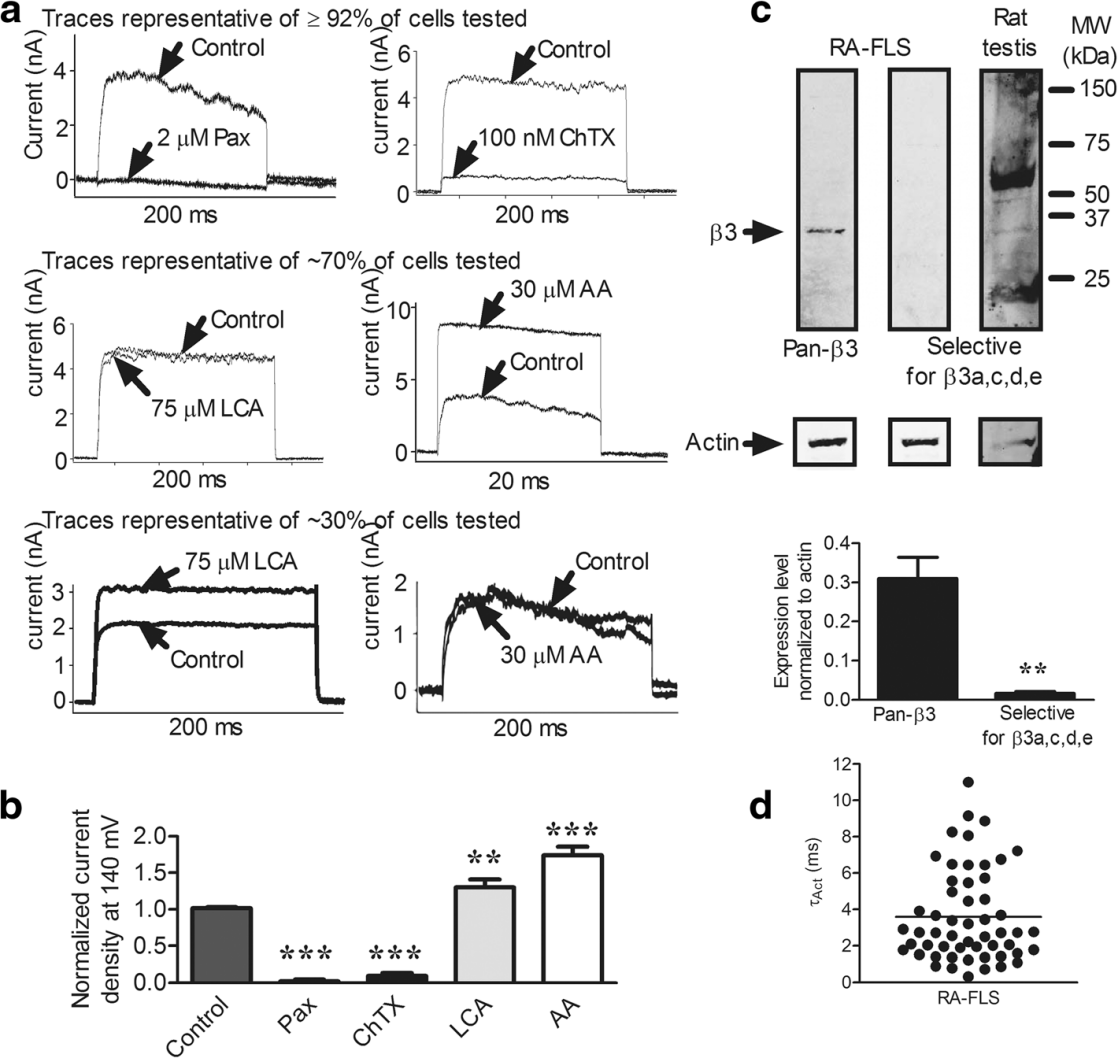
Functional KCa1.1 β3b subunits are present on the plasma membrane of fibroblast-like synoviocytes from patients with rheumatoid arthritis (RA-FLS). **a** Representative traces of whole-cell KCa1.1 currents elicited by 140-mV pulses for 200 milliseconds with 5 μM Ca^2+^ in the internal solution before (control) and after applying 2 μM paxilline (Pax), 100 nM charybdotoxin (ChTX), 30 μM arachidonic acid (AA), or 75 μM lithocholic acid (LCA). The two top traces are representative of ≥92 % of cells tested; the two middle traces are representative of approximately 70 % of cells tested; and the two bottom traces are representative of the other approximately 30 % of cells tested. **b** Peak KCa1.1 currents after different treatments normalized to the control levels. Mean ± SEM; *n* = 5 different donors. **c** Representative Western blot from a gel loaded with proteins from one RA-FLS donor or a rat testis extract. Different lanes were probed with antibodies against all splice variants of KCa1.1 β3 (pan-β3) or against KCa1.1 β3a, βc, βd, and βe only (*top*), and intensity of KCa1.1 β3 protein bands was normalized to actin expression levels in RA-FLS. Mean ± SEM; *n* = 6 different donors. **d** Activation kinetics (τ_Act_) of RA-FLS K^+^ currents. Each symbol on the scatterplot represents a different cell. *n* = 5 different donors; **p ≤ 0.01, ***p ≤ 0.001

The pore-forming α subunits of KCa1.1 can be detected at the plasma membrane and in the nucleus of RA-FLS [11]. Since our focus was on the channels expressed at the plasma membrane of FLS, and since coexpression of β subunits with α subunits affects the kinetics and pharmacology of K^+^ currents through the KCa1.1 channel, we used patch-clamp electrophysiology to assess the expression of functional β subunits at the plasma membrane of RA-FLS. Of the 51 cells from 5 different RA-FLS donors patch-clamped, 47 (92 %) exhibited a K^+^ current (Fig. 3a), as previously described [11]. Addition of paxilline, a blocker of the KCa1.1 α subunits regardless of β subunit expression [11, 14, 38], completely blocked the K^+^ current in all 14 cells tested (Fig. 3a, b), further confirming that the K^+^ channel observed was KCa1.1, as previously demonstrated [11]. The current displayed little or no inactivation in any of the cells tested (Fig. 3a). Since β2a, β3a, β3c, and β3e subunits have all been shown to induce partial or total inactivation of KCa1.1 [36, 39, 45–47], the lack of inactivation shows that these subunits are not involved in the KCa1.1 channel in RA-FLS.

To further identify the β subunits associated with the KCa1.1 channels in RA-FLS, we used KCa1.1 openers and blockers known to exert different effects on the channel, depending on its β subunit composition. We first tested the effects of the scorpion venom toxin ChTX on RA-FLS K^+^ currents, as ChTX can block only KCa1.1 channels that do not contain the β4 subunit [21, 36, 39]. ChTX inhibited the currents in all cells tested (Fig. 3a, b), demonstrating the absence of β4 subunits in KCa1.1 channels of RA-FLS. We next tested the effects of LCA, known to enhance currents through KCa1.1 channels only in the presence of β1 subunits [28, 36, 37]. An LCA-induced increase in current was observed in only 36 % of RA-FLS cells tested (Fig. 3a, b), demonstrating that KCa1.1 channels are formed of α and β1 subunits in approximately one-third of the cells. To identify the β subunit in the remainder of the RA-FLS, we used AA, known to open KCa1.1 channels and thus increase K^+^ currents in the presence of β2 and β3 subunits, but not β1 or β4 subunits [29, 36]. Such an increase was observed in 65 % of cells tested (Fig. 3a, b). Since the kinetics data above had already eliminated the possibility of β2a, β3a, β3c, or β3e subunits (Fig. 3a), this result with AA suggests that the majority of RA-FLS cells express a β3 subunit, either β3b or β3d. To discriminate between these two splice variants of β3, we performed Western blot experiments using two anti-β3 antibodies. The first antibody is directed to a conserved region of the β3 subunit, common to all five splice variants, and leads to a band of the correct molecular weight (Figs. 2, 3c). The second antibody used was raised against the N-terminus of β3 and therefore detects all splice variants of β3 other than β3b. Although this antibody detected a band of the correct molecular weight (about 32 kDa) in rat testis extracts, the only tissue with known β3 expression [26], it yielded no detectable band with RA-FLS extracts (Fig. 3c), suggesting that RA-FLS express the β3b subunit of KCa1.1. Since β1 but not β3b subunits slow the activation kinetics (τ_Act_) of KCa1.1 channels [48], we measured these kinetics in RA-FLS and found a spread in τ_Act_ with only 17 (31 %) of the 54 cells assessed having a τ_Act_ longer than 4 milliseconds and 69 % of cells having a τ_Act_ less than or equal to 4 milliseconds (Fig. 3d). These results reinforce the finding that different individual RA-FLS cells within a line express different β subunits.

### Expression of KCa1.1β3b is associated with higher levels of KCa1.1α and CD44

**Fig. 4:**
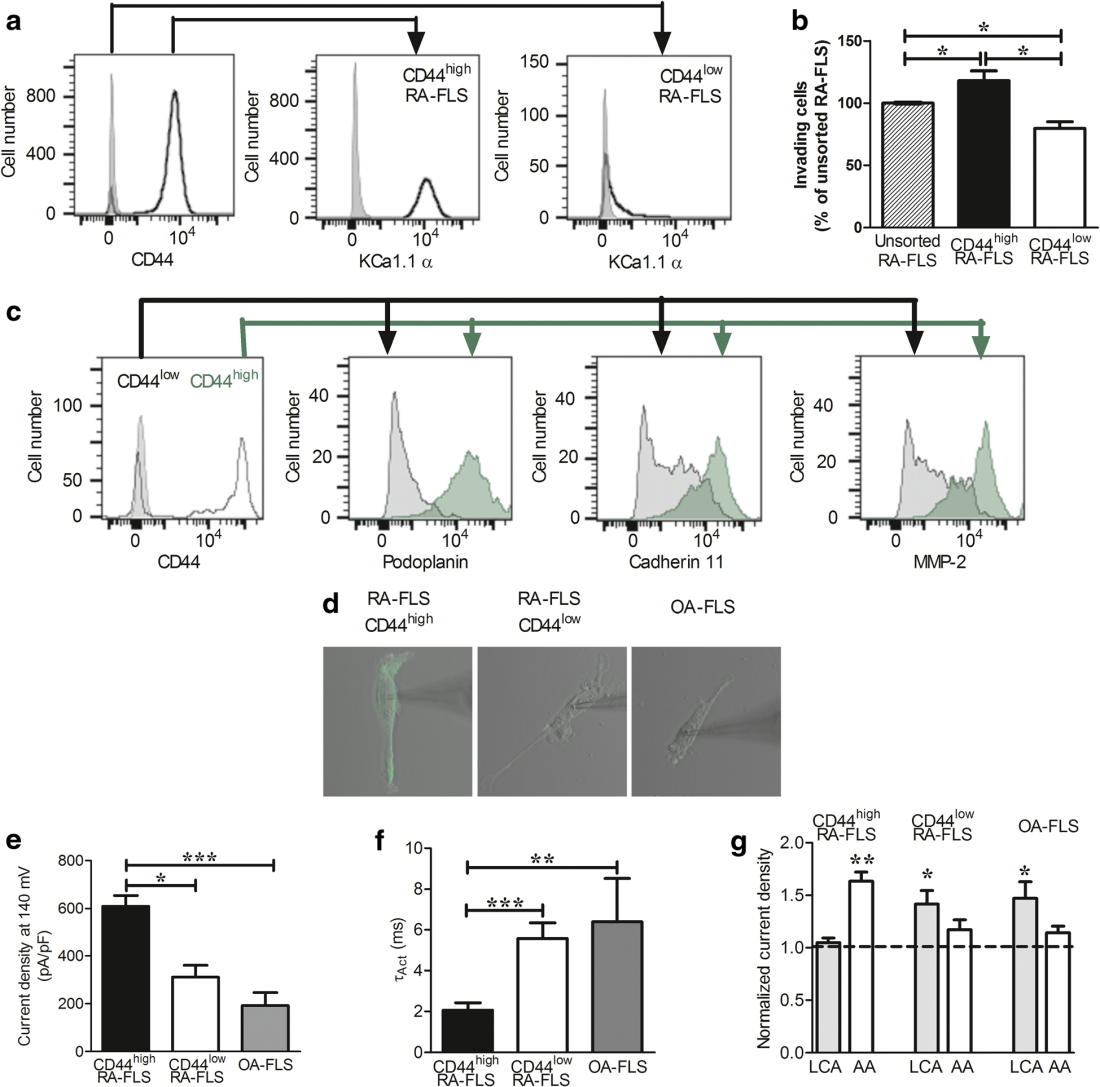
The calcium-activated potassium channel KCa1.1 β3 subunit is expressed by CD44^high^ fibroblast-like synoviocytes from patients with rheumatoid arthritis (RA-FLS), whereas the β1 subunit is expressed by CD44^low^ RA-FLS and fibroblast-like synoviocytes from patients with osteoarthritis (OA-FLS). **a** Representative flow cytometry histograms showing expression of KCa1.1α by CD44^high^ and CD44^low^ RA-FLS. Gray shading represents control staining; black lines represent CD44 (*left*) or KCa1.1α (*middle* and *right*) staining. **b** Invasiveness of unsorted RA-FLS and RA-FLS from the same donors sorted by flow cytometry into CD44^low^ and CD44^high^ populations. Mean ± SEM; *n* = 3 RA-FLS donors. The line for the error bar for the control was thickened compared with other plots to make it visible. **c** Representative flow cytometry histograms showing expression of podoplanin, cadherin-11, and matrix metalloproteinase (MMP)-2 by CD44^high^ (*green*) and CD44^low^ (*gray*) RA-FLS. In the *left panel*, gray shading represents control staining and *black lines* represent CD44 staining. **d** Individual RA-FLS stained for CD44. The patch-clamp pipette’s shadow is visible on the right of each image. **e** K^+^ current density elicited at 140 mV in CD44^high^ RA-FLS, CD44^low^ RA-FLS, and OA-FLS. Mean ± SEM; *n* = 5 RA-FLS donors and 4 OA-FLS donors. **f** Activation kinetics (τ_Act_) of K^+^ currents elicited at 140 mV in CD44^high^ RA-FLS, CD44^low^ RA-FLS, and OA-FLS. Mean ± SEM; *n* = 5 RA-FLS donors and 4 OA-FLS donors. **g** K^+^ current density after treatment of CD44^high^ RA-FLS, CD44^low^ RA-FLS, and OA-FLS with 75 μM lithocholic acid (LCA) or 30 μM arachidonic acid (AA) and normalized to current densities before treatment (*horizontal dashed line*). Mean ± SEM; *n* = 5 RA-FLS donors and 4 OA-FLS donors. *p ≤ 0.05, **p ≤ 0.01, ***p ≤ 0.001

Since invasiveness is an important feature of aggressive FLS during RA, we wanted to test whether invasiveness is associated with a differential expression of KCa1.1 β subunits by the cells. In the absence of antibodies that recognize an extracellular epitope of either β1 or β3 subunits, we searched for a surrogate marker with an extracellular epitope to allow for isolation of live cells. Elevated expression of CD44, a type I transmembrane glycoprotein that binds hyaluronan and other extracellular and cell surface ligands, by FLS and other cells was observed in RA [49, 50]. Interestingly, elevated CD44 expression is associated with enhanced invasiveness of cancer cells [51, 52]. To determine whether expression of β1 or β3 subunits is associated with CD44 expression levels and invasiveness in RA-FLS, we first showed an association between elevated expression of KCa1.1α and of CD44 (Fig. 4a). We next used flow cytometry to sort CD44^high^ and CD44^low^ RA-FLS and performed invasion assays. CD44^high^ RA-FLS were significantly more invasive than CD44^low^ cells (Fig. 4b). Since FLS invasiveness has also been associated with expression of podoplanin, cadherin-11, and MMP-2 [53, 54], we assessed expression levels of these three markers within the CD44^low^ and CD44^high^ populations of RA-FLS. We observed an association between elevated expression of CD44 and all three markers (Fig. 4c). We then stained cells for expression of CD44 and performed whole-cell patch-clamping to assess K^+^ current densities and τ_Act_ in CD44^high^ and CD44^low^ cells (Fig. 4d). CD44^high^ cells exhibited higher current densities and faster activation rates than did CD44^low^ cells, suggesting expression of β1 subunits by CD44^low^ cells and of β3b subunits by CD44^high^ cells (Fig. 4e, f). These results were further confirmed by assessing the effects of AA and LCA on the two cell subsets, as CD44^high^ cells displayed sensitivity to AA but not LCA, whereas CD44^low^ cells displayed sensitivity to LCA and not AA (Fig. 4g). As a control, we used minimally invasive FLS obtained from patients with OA [55]. OA-FLS exhibited low current densities at 140 mV, fast τ_Act_, and sensitivity to LCA but not to AA, similar to CD44^low^ RA-FLS (Fig. 4e–g).

### Knocking down the β3 subunit of KCa1.1 decreases cell surface expression of the pore-forming α subunit of KCa1.1

**Fig. 5:**
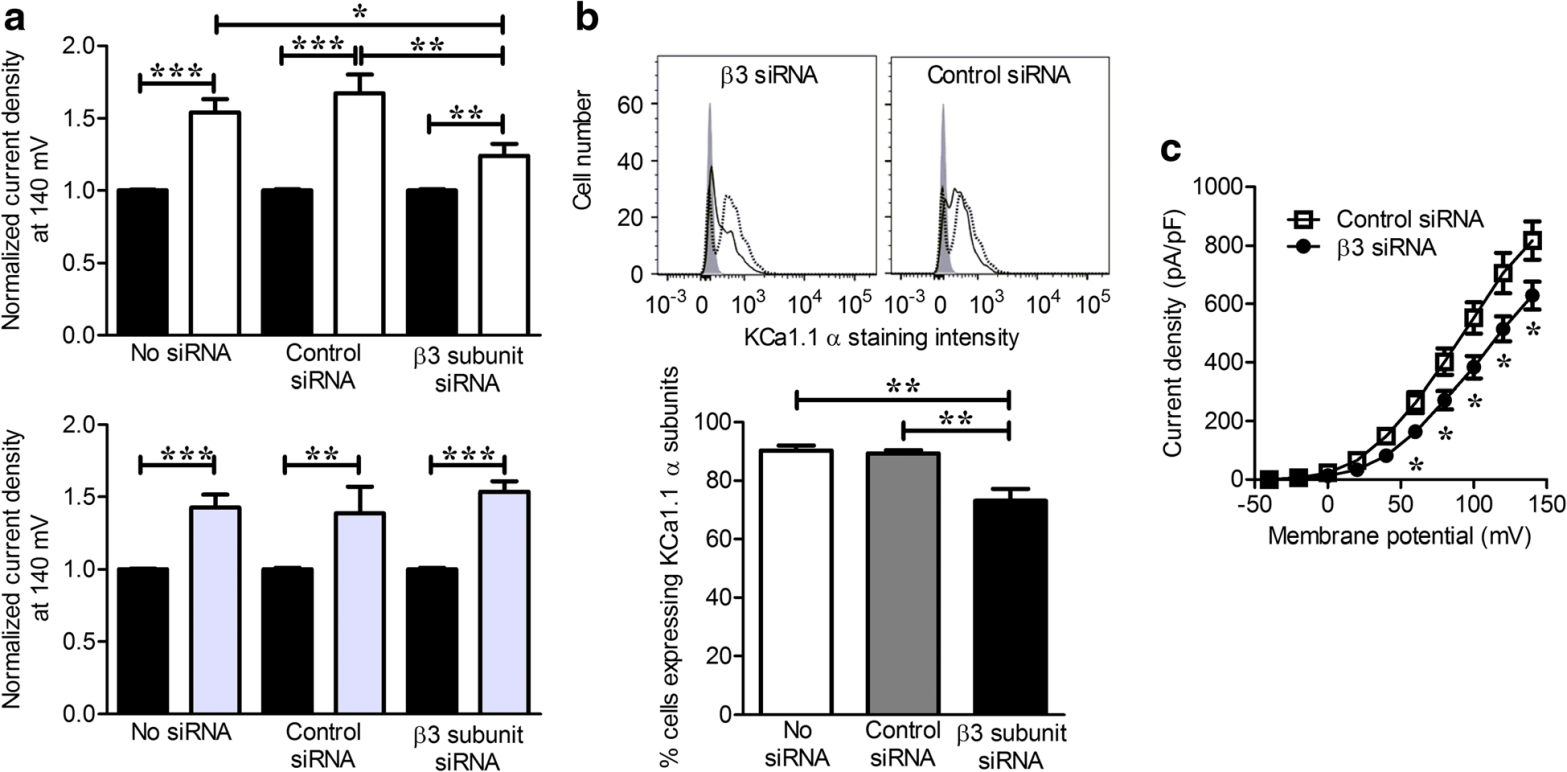
Silencing KCa1.1 β3 expression reduces cell surface expression of KCa1.1α. **a** K^+^ current density of untransfected fibroblast-like synoviocytes from patients with rheumatoid arthritis (RA-FLS) and RA-FLS transfected with control small interfering RNA (siRNA) or with siRNA against KCa1.1 β3 before (*black*) and after treatment with 30 μM arachidonic acid (AA) (*white*; *top plot*) or 75 μM lithocholic acid (LCA) (*gray*; *bottom plot*) and normalized to current densities before treatment. Mean ± SEM; *n* = 4 RA-FLS donors. **b** Representative flow cytometry histograms showing background staining (*gray shading*), expression levels of KCa1.1α in untransfected RA-FLS (*dashed line*) and RA-FLS transfected with siRNA against KCa1.1β3 (*solid black line*; *left histogram*) or with control siRNA (*solid black line*; *right histogram*). The bar graph below shows the percentage of cells expressing KCa1.1α calculated from the flow cytometric profiles of three RA-FLS samples. Mean ± SEM. **c** K^+^ current densities of RA-FLS transfected with control siRNA (*open boxes*) or with siRNA against KCa1.1β3 (*closed circles*) and pulsed stepwise from −40 to 140 mV in 20-mV increments. Mean ± SEM; *n* = 4 RA-FLS donors. *p ≤ 0.05, **p ≤ 0.01, ***p ≤ 0.001

We used a pool of siRNA to selectively inhibit gene expression of the β3 subunit. In whole-cell patch-clamp assays, RA-FLS became less sensitive to treatment with the β3 opener AA after transfection with β3, but not control siRNA, demonstrating the effectiveness of the siRNA. Neither control nor β3 siRNA affected the cells’ response to LCA, showing a lack of effect on β1 subunits (Fig. 5a). Flow cytometry measurements showed that β3 silencing induced a 20 % reduction in expression of the KCa1.1 α subunit (Fig. 5b). Moreover, whole-cell KCa1.1 current densities at voltages above 50 mV were significantly decreased after KCa1.1 β3 subunit silencing (Fig. 5c), suggesting lower surface expression of the pore-forming α subunit of KCa1.1.

### Knocking down the β3, but not the β1, subunit of KCa1.1 attenuates the ex vivo invasiveness of RA-FLS

**Fig. 6:**
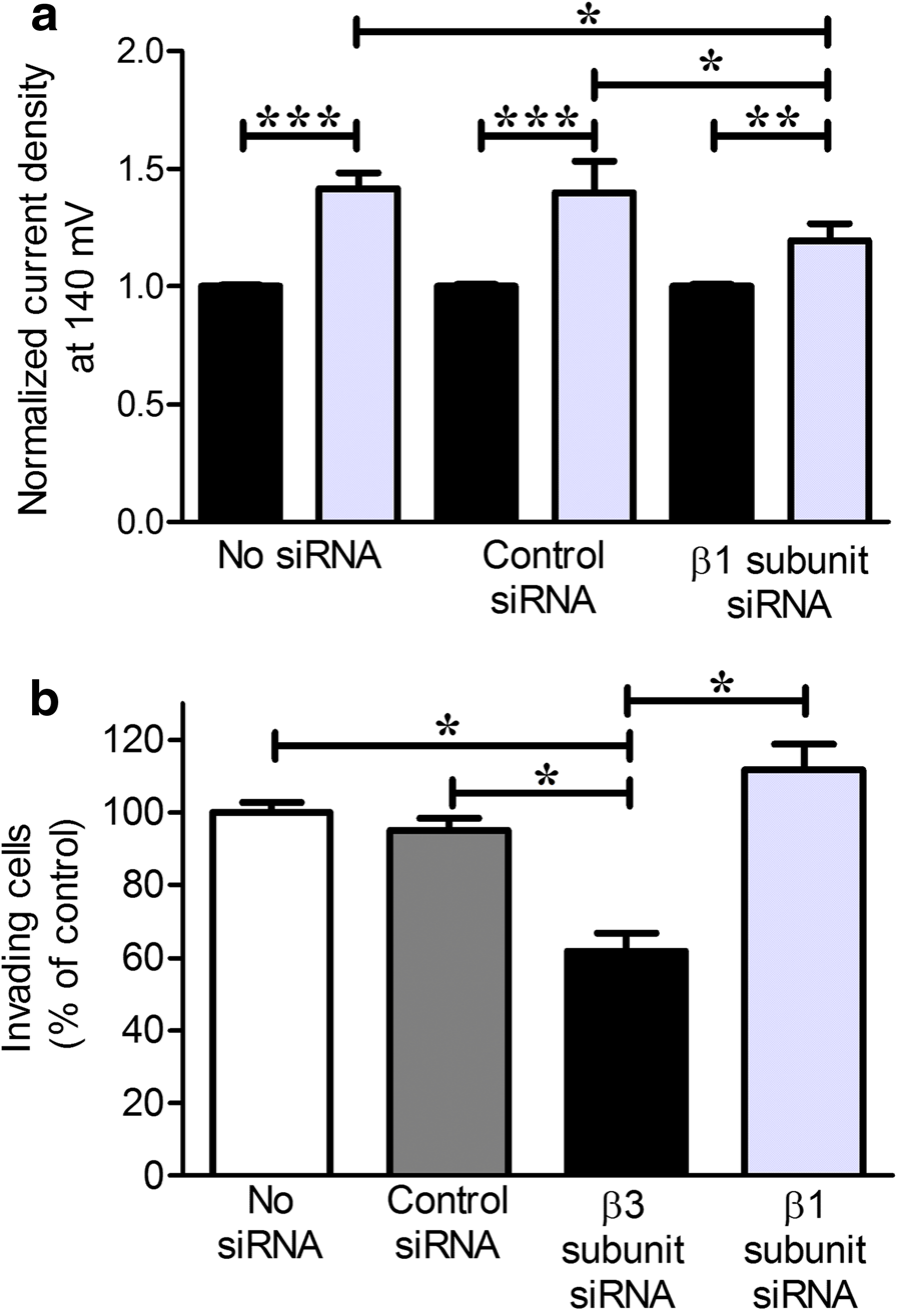
Silencing calcium-activated potassium channel KCa1.1β3 but not KCa1.1β1 expression reduces inhibits the invasiveness of fibroblast-like synoviocytes from patients with rheumatoid arthritis (RA-FLS). **a** K^+^ current density of untransfected RA-FLS and RA-FLS transfected with control small interfering RNA (siRNA) or with siRNA against KCa1.1 β1 before (*black*) and after treatment with 75 μM lithocholic acid (*gray*) and normalized to current densities before treatment. Mean ± SEM; *n* = 3 RA-FLS donors. **b** Invasiveness of untransfected RA-FLS and RA-FLS transfected with control siRNA, with siRNA against KCa1.1 β3, or with siRNA against KCa1.1 β1. Mean ± SEM; *n* = 4 RA-FLS donors. *p ≤ 0.5, **p ≤ 0.01, ***p ≤ 0.001

Reducing the expression or function of the α subunit of KCa1.1 inhibits the invasiveness of FLS [11, 12]. We therefore assessed the effects of silencing the β3 subunit of KCa1.1 on RA-FLS invasiveness using Matrigel invasion assays. We also used a pool of siRNA to inhibit the expression of the β1 subunit of KCa1.1 and used the channel’s sensitivity to LCA to demonstrate the effectiveness of the siRNA, as control siRNA did not affect the RA-FLS response to LCA, whereas β1 siRNA reduced it (Fig. 6a). Whereas β3 siRNA reduced the invasiveness of RA-FLS, silencing the β1 subunit of KCa1.1 did not affect this invasiveness (Fig. 6b).

## Discussion

The KCa1.1 channel has been proposed as a therapeutic target in the treatment of RA [11, 12]. However, the wide tissue distribution of the pore-forming α subunit of the channel precludes the use of blockers targeted to this subunit alone, owing to the risk of severe side effects in multiple organ systems [16–18]. KCa1.1 does, however, remain an attractive target for therapy because the regulatory β subunits of KCa1.1 have restricted tissue distribution [20, 22]. In the present study, we demonstrated that RA-FLS express functional β1 and β3 subunits of KCa1.1 at their plasma membrane and that expression of β3 is higher on CD44^high^ RA-FLS and is associated with a higher expression level of KCa1.1α. Silencing β3, but not β1, significantly reduced the invasiveness of RA-FLS. In addition, silencing β3 reduced the expression level of KCa1.1α.

Analysis by qRT-PCR showed expression of most β subunits in RA-FLS at the mRNA level. Low mRNA expression levels of β accessory subunits have been described in the majority of tissues; therefore, it was not surprising to find similar results in RA-FLS [26]. In addition, mRNAs are not always translated into proteins, requiring the detection of the proteins themselves. In Western blotting experiments, we detected β3 subunits in all samples analyzed; however, other β subunits were also detectable at the mRNA and protein levels in some samples. Determination of the kinetics and pharmacological profile of KCa1.1 currents of RA-FLS by single-cell electrophysiology demonstrated the expression of functional β1 and β3 subunits at the plasma membrane. Previous work has also shown the expression of the α subunit of KCa1.1 in the nucleus of RA-FLS and in several organelles in different cell types [56]. This raises the possibility that β subunits of the channel may also be expressed by organelles and play a role not yet understood in FLS.

Levels of the β subunits were lower than those of the α subunit as measured at the mRNA and protein levels by qPCR and Western blot analysis, respectively. It is therefore likely that each α subunit tetramer is associated with fewer than four β subunits. The ratio of β and α subunits required to modify current kinetics and pharmacological responses of KCa1.1 channels remains unclear. Our electrophysiology results demonstrate that the numbers of β1 or β3 expressed by RA-FLS are sufficient to affect the function of the KCa1.1 channels at their plasma membrane.

The majority of RA-FLS had characteristic whole-cell KCa1.1 K^+^ currents sensitive to paxilline, except for approximately 8 % of the cells that displayed no K^+^ currents under the conditions used, confirming our previous study findings [11]. No single assay is sufficient to identify the β subunits associated with α subunits to form KCa1.1 channels. We therefore used a combination of patch-clamp electrophysiology to measure activation and inactivation kinetics of the currents and test the effects of well-characterized pharmacological agents and of Western blots using antibodies raised against different epitopes of the β3 subunit. When examining the pharmacological response to the perfused agents, we used the bile acid derivate LCA, which, at concentrations of 50–150 μM acts as a potent reversible potentiator of KCa1.1 currents only in the presence of β1 subunits [27, 28]. A minority of cells responded to LCA, indicating functional β1 subunit expression on some RA-FLS. In contrast, treating the RA-FLS with 30 μM AA, known to enhance KCa1.1 currents only in the presence of β2 or β3 subunits [29], induced an increase in current amplitude in the majority of the cells. We tested ChTX, which blocks KCa1.1 channels associated with β1, β2, and β3, but not β4 subunits [21], leading to current block in 100 % of the RA-FLS assayed and thus demonstrating the absence of β4 subunits as components of KCa1.1 at the plasma membrane of these cells. A phenotype of noninactivating KCa1.1 currents blocked by both paxilline and ChTX, and potentiated by AA but not by LCA, leads to the conclusion that the majority of RA-FLS mainly express functional KCa1.1 α and β3 subunits. Western blots using antibodies specific to different β3 epitopes indicated that RA-FLS express the β3b isoform.

Our data show an association between the expression of KCa1.1 channels formed of α and β3 subunits, high current densities, and a high expression of CD44, previously shown in RA synovial tissues [49, 50], whereas the αβ1 phenotype was associated with low expression of CD44 and lower current densities. Since we had previously shown increased expression of KCa1.1α in invasive FLS [12], this raised the possibility that a differential expression of β subunits could underlie the different expression levels of the α subunit of KCa1.1. Indeed, silencing of the β3 subunit did decrease expression levels of KCa1.1α and reduced the K^+^ current densities, suggesting that β3 may participate in the cell surface expression of the channel, as has been described in other systems with β1, β2, and β4 [23, 24, 30].

To our knowledge, an association between high expression levels of a potassium channels and CD44 has not directly been reported. However, CD44 expression has long been associated with cancer cell metastasis [52]. More recently, the expression of various potassium channels, including KCa1.1, has also been linked to enhanced metastatic potential in cancer [57]. Further work needs to be done to determine whether CD44 and potassium channels share any signaling pathways leading to their concomitant upregulation in highly motile cells and to assess whether their expression or function is interdependent in these cells. Additional work is also required to determine whether the switch from KCa1.1 β1 to β3b expression in RA-FLS is a consequence, an initiating event, or an independent event in the upregulation of CD44 by these cells. Finally, it will be interesting to establish whether KCa1.1 and CD44 play synergistic roles in regulating the invasiveness of RA-FLS.

The work presented here was focused on the regulatory β subunits of KCa1.1. In the last few years, γ subunits of KCa1.1 have also been identified in various tissues [25, 58]. These subunits are structurally different from β subunits, but, like β subunits, they affect the function of the KCa1.1 channels with which they are coexpressed. It is possible that FLS express γ subunits of KCa1.1 in addition to the β subunits we have identified, either at the plasma membrane or in organelles.

The KCa1.1 channel formed of α and β3 subunits expressed by RA-FLS represents an attractive therapeutic target for RA. The mRNA for β3 is expressed only in very low quantity in most tissues, with the highest levels detected in the testis [26]. The incidence of RA is higher in women than in men [1–3]; in the majority of patients, there would therefore not be a concern about male reproductive organ toxicity. Furthermore, the testes are protected by the blood-testis barrier that prevents access by many drugs [59]. It is therefore possible to design KCa1.1 blockers that cannot cross this barrier.

In addition to this restricted tissue distribution, the presence of β subunits alters the pharmacology of the channel. Indeed, several scorpion venom peptides affect KCa1.1 channels differently, depending on the β subunit expressed. ChTX and iberiotoxin both block KCa1.1 channels containing β1, β2, and β3 subunits, but not β4 subunits, [21, 60]. Martentoxin blocks KCa1.1 formed of αβ4, whereas it opens KCa1.1 formed of αβ1 and has yet to be tested on KCa1.1 containing β2 or β3 subunits [61]. It is therefore conceivable that venom peptides that selectively target KCa1.1 αβ3 could be identified or that existing KCa1.1-blocking peptides could be engineered to enhance their selectivity for this channel as was done successfully with other K^+^ channel-blocking peptides [62–64].

## Conclusions

We provide evidence that the KCa1.1 channels expressed at the plasma membrane of human FLS contain regulatory β1 or β3b subunits. Expression of the β3b subunit is associated with a highly invasive behavior of FLS and high expression of CD44, whereas the β1 subunit is associated with low levels of CD44 and lower invasiveness of the cells. These data suggest that a blocker selective for KCa1.1 channels formed of α and β3b subunits and that cannot cross the blood-testis barrier will be attractive for targeting KCa1.1 channels on invasive RA-FLS for the treatment of RA.

## Abbreviations

AA: arachidonic acid
cDNA: complementary DNA
ChTX: charybdotoxin
ΔcT: comparative cycle threshold
DMARD: disease-modifying antirheumatic drug
DMSO: dimethyl sulfoxide
EP: electrophysiology
FC: flow cytometry
FITDP: Feinstein Institute Tissue Donation Program
FLS: fibroblast-like synoviocyte(s)
GAPDH: glyceraldehyde 3-phosphate dehydrogenase
IgG: immunoglobulin G
IRB: institutional review board
KCa: calcium-activated potassium channel
LCA: lithocholic acid
MMP: matrix metalloproteinase
mRNA: messenger RNA
N/A: not available
NSAID: non-steroidal anti-inflammatory drug
OA: osteoarthritis
OA-FLS: fibroblast-like synoviocytes from patients with osteoarthritis
RA: rheumatoid arthritis
RA-FLS: fibroblast-like synoviocytes from patients with rheumatoid arthritis
RF: rheumatoid factor
RT: reverse transcription
siRNA: small interfering RNA
τ_Act_: activation kinetics
WB: Western blot
