# Erratum to: Different expression of β subunits of the KCa1.1 channel by invasive and non-invasive human fibroblast-like synoviocytes

**DOI:** 10.1186/s13075-016-1024-z

**Published:** 2016-06-01

**Authors:** Zoltán Pethő, Mark R. Tanner, Rajeev B. Tajhya, Redwan Huq, Teresina Laragione, Gyorgy Panyi, Pércio S. Gulko, Christine Beeton

**Affiliations:** Department of Molecular Physiology and Biophysics, Mail Stop BCM335, Room S409A, Baylor College of Medicine, Houston, TX 77030 USA; Department of Biophysics and Cell Biology, Faculty of Medicine, University of Debrecen, Debrecen, 4032 Hungary; Interdepartmental Graduate Program in Translational Biology and Molecular Medicine, Baylor College of Medicine, Houston, TX 77030 USA; Graduate Program in Molecular Physiology and Biophysics, Baylor College of Medicine, Houston, TX 77030 USA; Division of Rheumatology, Icahn School of Medicine at Mount Sinai, New York, NY 10029 USA; Biology of Inflammation Center, Baylor College of Medicine, Houston, TX 77030 USA; Center for Drug Discovery, Baylor College of Medicine, Houston, TX 77030 USA

After publication of this article [1], it was noticed that the Ethical Considerations subsection within the Methods section was worded such that it suggested that the Feinstein Institute Tissue Donation Program collected synovial tissues and isolated FLS. For clarity, the Ethical Considerations statement should instead be read as, “De-identified synovial samples were collected for research under the approval of the Institutional Review Board (IRB) of the Feinstein Institute for Medical Research. All patients provided written consent to have their tissues, RNA, DNA, and cells studied. The consent forms are held by the Feinstein Institute Tissue Donation Program; Dr. Gulko's group isolated and characterized the FLS and banked them for research. The IRB at Baylor College of Medicine has established that the work conducted there did not constitute human research since the samples were already banked for research and were de-identified; thus could not be traced back to their donor.”
